# RNA modification patterns based on major RNA modifications define tumor microenvironment characteristics in glioblastoma

**DOI:** 10.1038/s41598-022-14539-6

**Published:** 2022-06-18

**Authors:** Ganglei Li, Yu Zhu, Jun Gu, Tiesong Zhang, Feng Wang, Kaiyuan Huang, Chenjie Gu, Kangli Xu, Renya Zhan, Jian Shen

**Affiliations:** Department of Neurosurgery, The First Affiliated Hospital, College of Medicine, Zhejiang University, No.79 Qingchun Road, Hangzhou, 310003 Zhejiang China

**Keywords:** CNS cancer, Tumour biomarkers, Biomarkers

## Abstract

RNA modifications play a major role in tumorigenicity and progression, but the expression and function in glioblastoma (GBM) have not been well described. In this study, we developed a GBM score based on the differentially expressed genes (DEGs) between groups showing RNA modification patterns. We assessed the association between the GBM score and tumor microenvironment (TME) characteristics. Based on the gene expression of these regulators, we identified two clusters with distinct RNA modification patterns. Kaplan–Meier survival curves showed that patients in cluster 1 had worse survival than those in cluster 2. Kaplan–Meier and multivariate Cox regression analyses showed that GBM scores (based on DEGs between RNA modification patterns) are an independent predictive biomarker for patient prognosis. Besides, we found that samples with high scores were significantly associated with epithelial-to-mesenchymal transition and immune checkpoints, while samples with low scores were associated with cell cycle regulation. Importantly, GBM-score markedly positively correlated drug resistance, while negatively correlated with drug sensitive. The responders of anti-PD-1/PD-L1 immunotherapy tend to have a lower GBM score than non-responders. In conclusion, our comprehensive analysis of multiple RNA modifications in GBM revealed that RNA modification regulators were closely correlated with TME.

## Introduction

Glioblastoma (GBM) is one of the most common and aggressive intracranial tumors in adults, comprising 45.6% of all primary malignant brain cancers^1^. The 5-year survival rate of patients with GBM is only 5%, and the median overall survival time is just 12–15 months^2^. GBM is an extremely aggressive tumor with a high frequency of recurrence after surgery, radiotherapy, and chemotherapy^2^. Further, GBM is a highly heterogeneous neoplasm that displays a wide range of genetic and epigenetic changes^3^. A better understanding of the molecular mechanisms that underlie GBM may lead to more effective prognostic markers and therapeutic targets for GBM.

RNA modification is an important method of posttranscriptional gene regulation that affects many biological processes^4,5^. The most common modification of mRNA is the addition of 6-methyladenosine (m6A), which regulates the biogenesis and function of non-coding RNAs, including long non-coding RNAs, microRNAs, and circular RNAs^6^. Three types of RNA modification regulators have been identified: “writers” (which add a specific modification), “erasers” (which remove a specific modification), and “readers” (which identify and bind modified nucleotides). Most RNA modifications are the result of writers. There is increasing evidence that there are multiple regulators for m6A modification that are important in tumor initiation, progression, immune modulation, and effective treatments^7^. The role of other RNA modifications, including 1-methyladenosine (m1A), Alternative polyadenylation (APA), RNA editing, 5-methylcytosine (m5C), and pseudouridine (Ψ), are poorly understood^8,9^.

RNA modifications, especially m6A addition, influence the diversity and complexity of the tumor microenvironment (TME) and immune cell-infiltrating characteristics of the tumor^6^. Zhang et al. reported that m6A modification patterns were predictive for prognosis, inflammation status, TME phenotype, and genetic variation^10^. Particular m6A modification patterns are linked to successful immunotherapy, guiding immunotherapy strategies^10^. Based on RNA m6A regulators, Lin et al. constructed a prognostic model for glioma that provides insight into the relationship between m6A modification and the infiltration of immune cells^11^. However, it is difficult to evaluate the role of RNA modification in cancers because different RNA methylation modifications interact; therefore, the study of a single type of RNA modification in tumors is not optimal.

In this study, we explored the expression profile of RNA modification writers using data from The Cancer Genome Atlas (TCGA). Based on the expression of those regulators, we identified two distinct gene expression patterns of RNA modification writers(cluster 1 and cluster 2) using unsupervised clustering analysis. Both clusters were significantly correlated with differences in immune cell infiltration and prognosis of GBM patients. We developed a scoring model based on the differentially expressed genes (DEGs) between cluster 1 and cluster 2 to assess RNA modifications for individual patients. Here, we demonstrate the value of this scoring model in epithelial-to-mesenchymal transition (EMT) characteristics, drug sensitivity, and response to immunotherapy.

## Results

### Alterations of RNA modification writer genes in GBM

**Figure 1:**
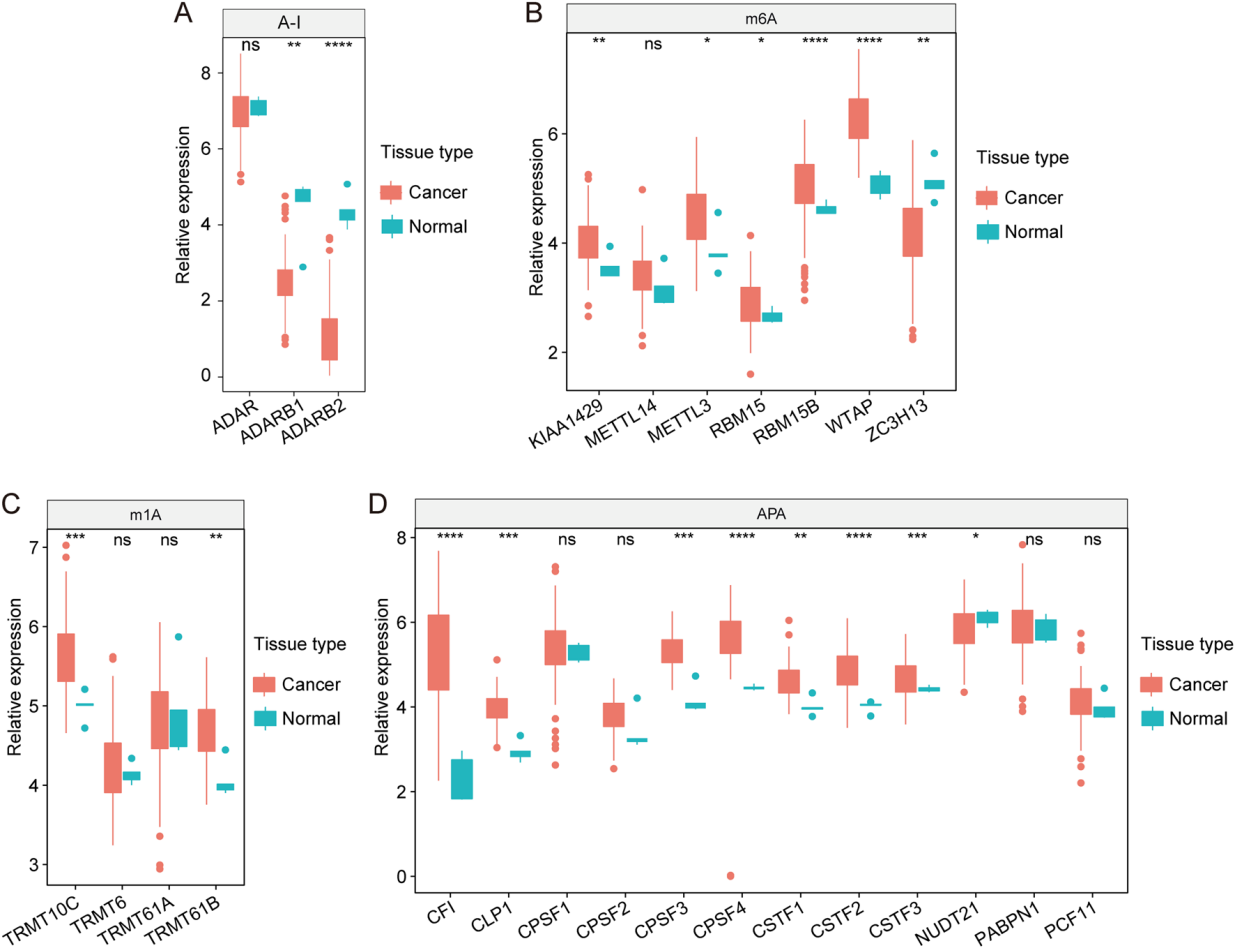
The mRNA expression of 26 writers for four types of RNA modifications in tumor vs. normal tissue. (**A**–**D**) The expression differences of writers of A-to-I RNA editing, m6Am, m1A, and APA in tumors and normal samples are shown.

In this study, we focused on the four main types of RNA modifications (m1A methylation, m6A methylation, APA, and A-to-I RNA editing) and identified 26 RNA modification writer genes (Supplementary Table S1). We analyzed the expression of the 26 regulators in tumor versus adjacent tissue from 167 GBMs and 5 normal sample cohorts. We found that 15 of the 26 regulators were more highly expressed in tumor tissue than adjacent tissue (Fig. 1A–D). These results demonstrated high levels of heterogeneity in gene expression in GBM, suggesting that the RNA regulators play a vital role in GBM pathogenesis and progression.

### Distinct RNA modification patterns mediated by RNA modification regulators

**Figure 2:**
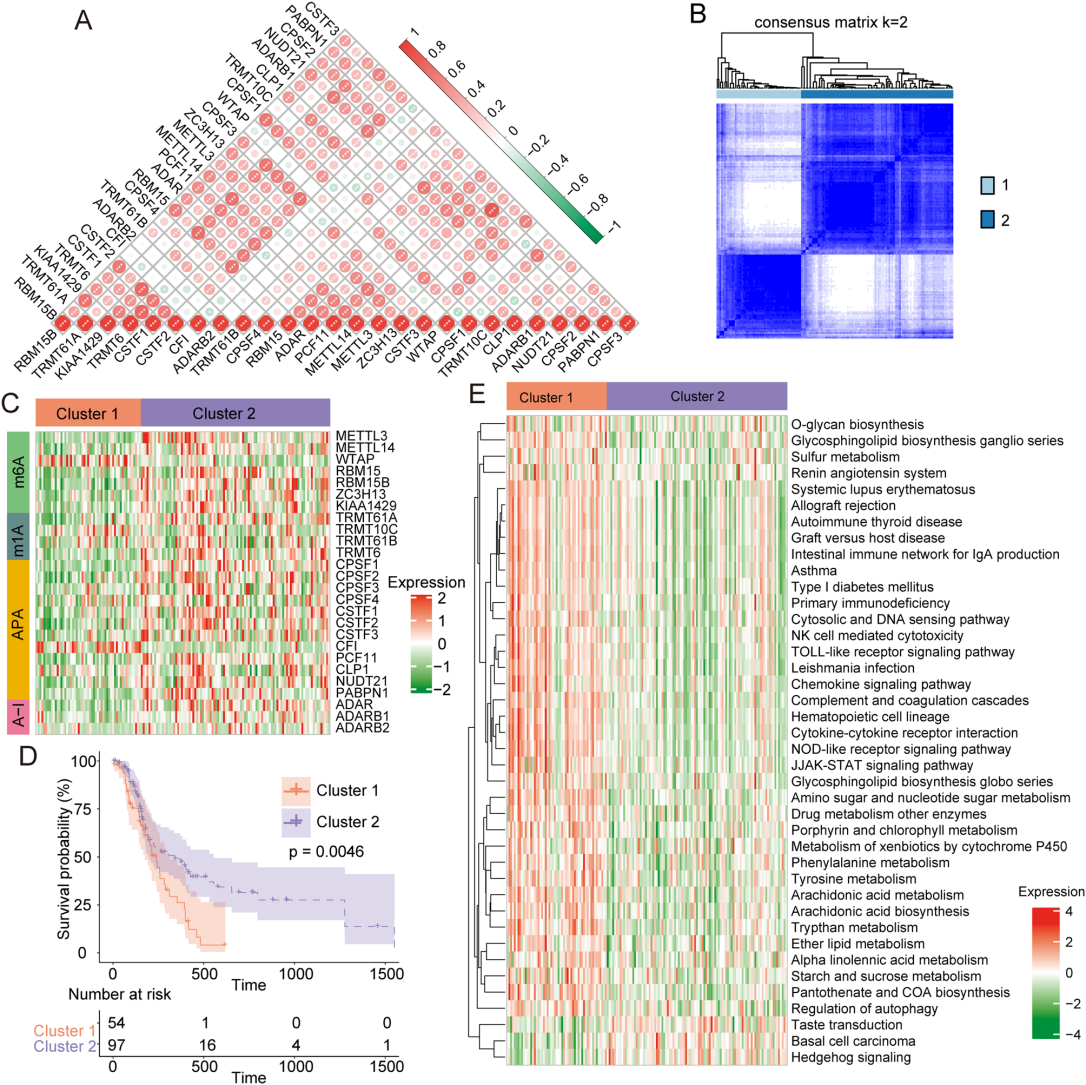
RNA modification patterns and related biological pathways. (**A**) Heatmap showing the correlation among the 26 RNA modification regulators in GBM. (**B**) The consensus map of NMF was used for subtype analysis. (**C**) Consensus clustering algorithm to identify distinct RNA modification patterns. (**D**) The difference in prognosis between two RNA modification patterns. (**E**) Enrichment analysis shows the related biological pathways in cluster 1 and cluster 2.

To evaluate crosstalk among the 26 RNA modification regulators, we assessed the correlation among these writers using Spearman’s correlation analysis. Positive correlations were found among several writers (Fig. 2A), indicating that crosstalk among the writers might mediate the formation of distinct RNA modification patterns and GBM progression. Thus, we used consensus clustering analysis to identify the different RNA modification patterns based on the expression of the 26 writer genes. We choose K = 2 as the final number of clusters, according to the smallest clustering within groups (Fig. 2B). Two RNA modification clusters were found, clusters 1 and 2 (Fig. 2C). Based on Kaplan–Meier survival curves, patients in cluster 1 had lower survival than those in cluster 2 (Fig. 2D). To identify potential changes in molecular pathways among these RNA modification patterns, we used GSVA to estimate the variation in gene set enrichment. Cluster 1 was significantly enriched in various signaling pathways, such as those involved in NK cell-mediated cytotoxicity, Toll-like receptor activation, JAK-STAT, and chemokine signaling (Fig. 2E). The enriched signaling pathways of cluster 2 were significantly correlated with immune activation, including regulation of autophagy, taste transduction, basal cell carcinoma, and hedgehog signaling (Fig. 2E). These results suggest that RNA modification patterns correlate with important biological behaviors in GBM.

### Immune cell infiltration characteristics in RNA modification patterns

**Figure 3:**
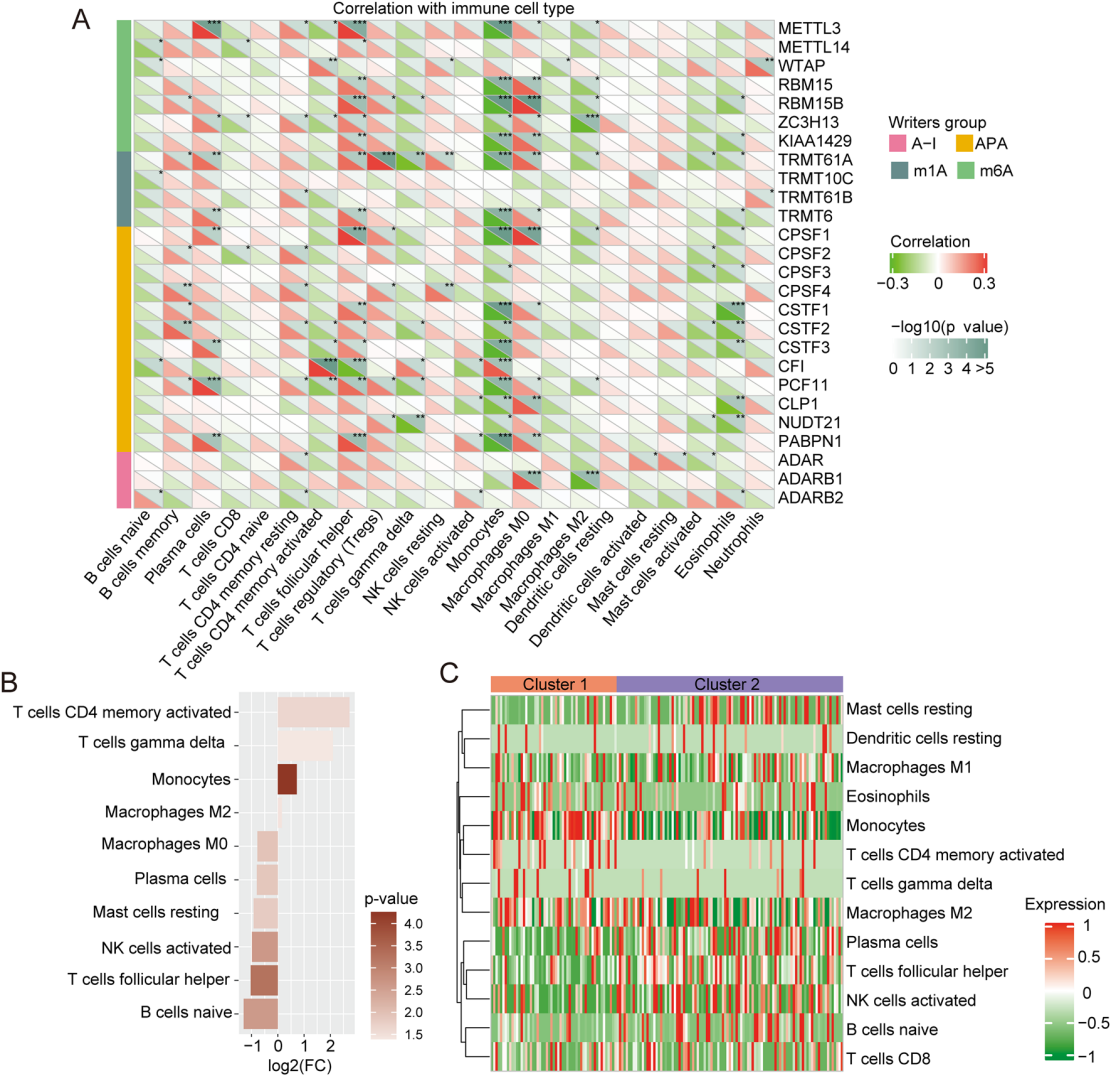
Immune cell infiltration characteristics for different RNA modification patterns. (**A**) The correlation between 26 RNA modification regulators and different types of immune cells in GBM. (**B**) CIBERSORT was used to compare the relative abundance of infiltrating immune cells between cluster 1 and cluster 2. Difference < 0 shows that the infiltrating immune cells are mainly enriched in cluster 2. (**C**) The different types of immune cells were analyzed by ssGSEA in cluster 1 versus cluster 2.

Because regulators of m6A methylation modification shape the diversity and complexity of the TME^10–13^, we analyzed the role of RNA modification in the TME using CIBERSORT. We found that RNA modification regulators RBM15, RBM15B, TRMT6, CLTP1, PABPN1, ADARB1, and CPSF1 were markedly positively associated with M0 macrophage differentiation (Fig. 3A). To further determine the immune cells characteristics of the distinct RNA modification patterns, we calculated the ssGSEA score of the immune cells. Although the expression plots of immune cells seemed highly heterogeneous in cluster 1 and cluster 2, immunosuppressive cells, such as M2 macrophages tend to be higher in cluster 1 than cluster 2, while M1 macrophages, activated NK cells, and plasma cells tend to be more enriched in cluster 2 (Fig. 3B,C). These results indicated that RNA modification patterns were closely correlated with the TME cell infiltration characteristics of GBM.

### Generation of an RNA modification regulator model

**Figure 4:**
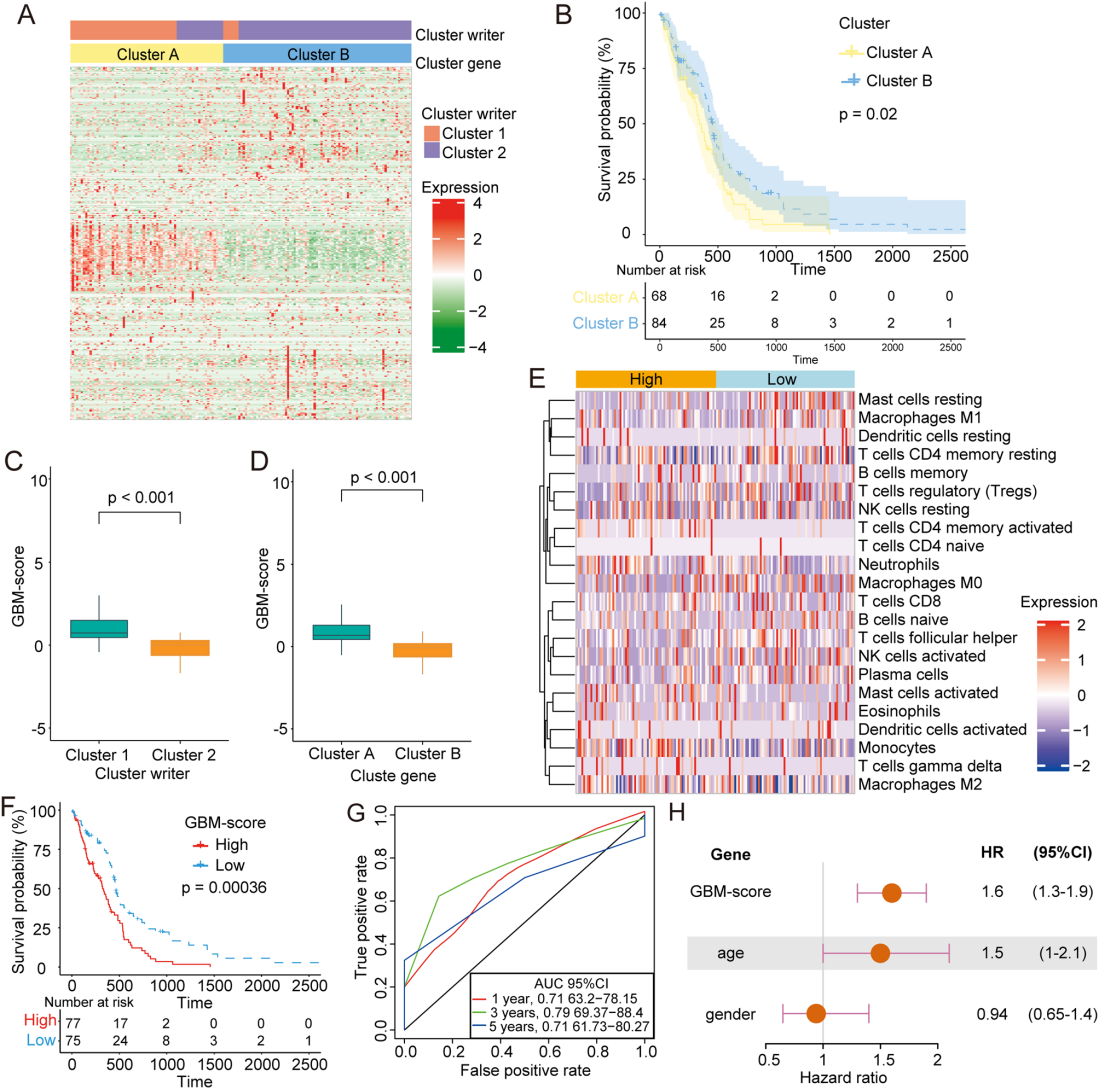
Generation of the RNA modification regulator model. (**A**) An unsupervised clustering method was used to classify GBM patients into gene subgroups based on the DEGs of distinct RNA modification patterns. (**B**) Kaplan–Meier curves for cluster A and cluster B. (**C**,**D**) The Wilcoxon test was performed to assess differences in the GBM scores between distinct RNA modification patterns and gene-subgroups. (**E**) The ssGSEA score of immune cells in high and low GBM score groups. (**F**) The survival analysis of patients with GBM scores in high and low subgroups. (**G**) The AUC for the GBM score for patients with 365 days, 1095 days, or 1825 days overall survival. (**H**) Multivariate Cox regression model analysis in the TCGA-GBM cohorts.

While attempting to elucidate the regulatory mechanisms for RNA modifications, we found 343 DEGs involved in RNA modification between the two RNA modification patterns (cluster 1 and cluster 2). Based on these DEGs, we classified GBM patients into distinct gene subgroups using an unsupervised clustering method and identified gene subgroup clusters A and cluster B (Fig. 4A). Kaplan–Meier analysis showed that patients in cluster A were significantly correlated with a poor prognosis compared to patients in cluster B (Fig. 4B). The prognosis of patients in cluster A was similar to patients in cluster 1 (Fig. 2C).

The analysis of RNA modification patterns was based only on patient populations. Given the heterogeneity of RNA modifications in individuals, we developed a scoring model to evaluate the RNA modification pattern of individual patients with GBM, which we termed the GBM score model. Cluster 2 had a significantly lower median score compared to cluster 1, and cluster B had a lower median score compared to cluster A (Fig. 4C,d). We suspected that the GBM score was associated with immune cell infiltration in the TME, so we compared the abundance of different types of immune cells between high and low GBM score groups. The infiltration rate of monocytes and CD8 + T cells was higher in the high GBM score group, while the infiltration rate of activated NK cells and M1 macrophages was higher in the low GBM score group (Fig. 4E). To better illustrate the utility of the GBM score model, we also determined the correlation between prognosis and GBM scores. In TCGA cohort, Kaplan–Meier analysis showed that patients in the high-score group had a worse prognosis than those in the low-score group (Fig. 4F). The areas under the curve (AUC) for the GBM scores were 0.71, 0.79, and 0.71 at 365 days, 1095 days, and 1825 days overall survival, respectively (Fig. 4G). The results of multivariate Cox regression analysis showed that the GBM score was an independent biomarker for evaluating patient prognosis (Fig. 4H; HR = 1.6, 95% confidence interval 1.3–1.9, *P* < 0.001).Thus, the GBM score can quantify the RNA modification pattern and may serve as a predictor for the prognosis for patients with GBM.

### GBM score and EMT characteristics

**Figure 5:**
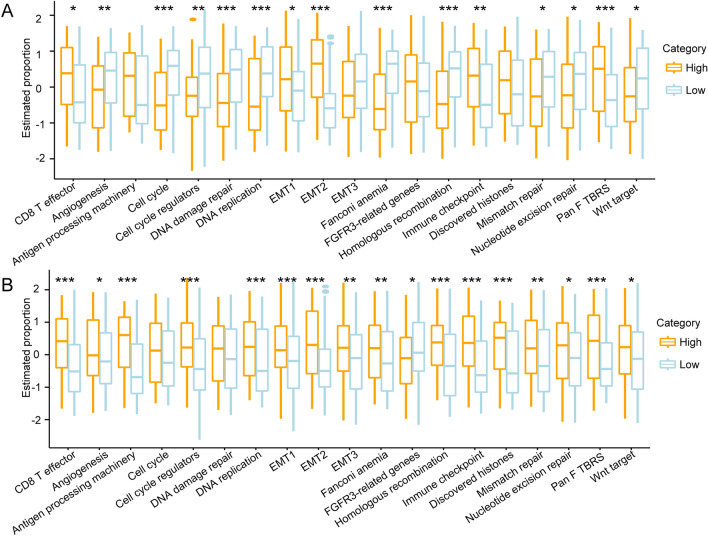
GBM score and EMT characteristics. (**A**,**B**) The method ssGSEA was used to analyzed the difference in EMT-related signaling pathways between low and high GBM score groups in TCGA-GBM datasets and the CGGA cohort**.**

EMT is a fundamental cellular process that plays a vital role in embryonic development^14^. In cancer, reactivation of the EMT enhances the metastatic properties of tumor cells, including invasion, migration, drug resistance, and tumor initiation potential^14^. To determine the association between GBM score and clinical characteristics, we analyzed the characteristics of the EMT-related pathways in the high and low GBM score groups. The two groups of samples with different GBM scores had different pathway characteristics. In the TCGA dataset, samples with high scores were significantly associated with EMT and immune checkpoints, while samples with low scores were associated with cell cycle regulation (Fig. 5A). Similar results were observed for the Chinese Glioma Genome Atlas (CGGA) datasets (Fig. 5B).

### Drug sensitivity differences between low and high GBM score groups

**Figure 6:**
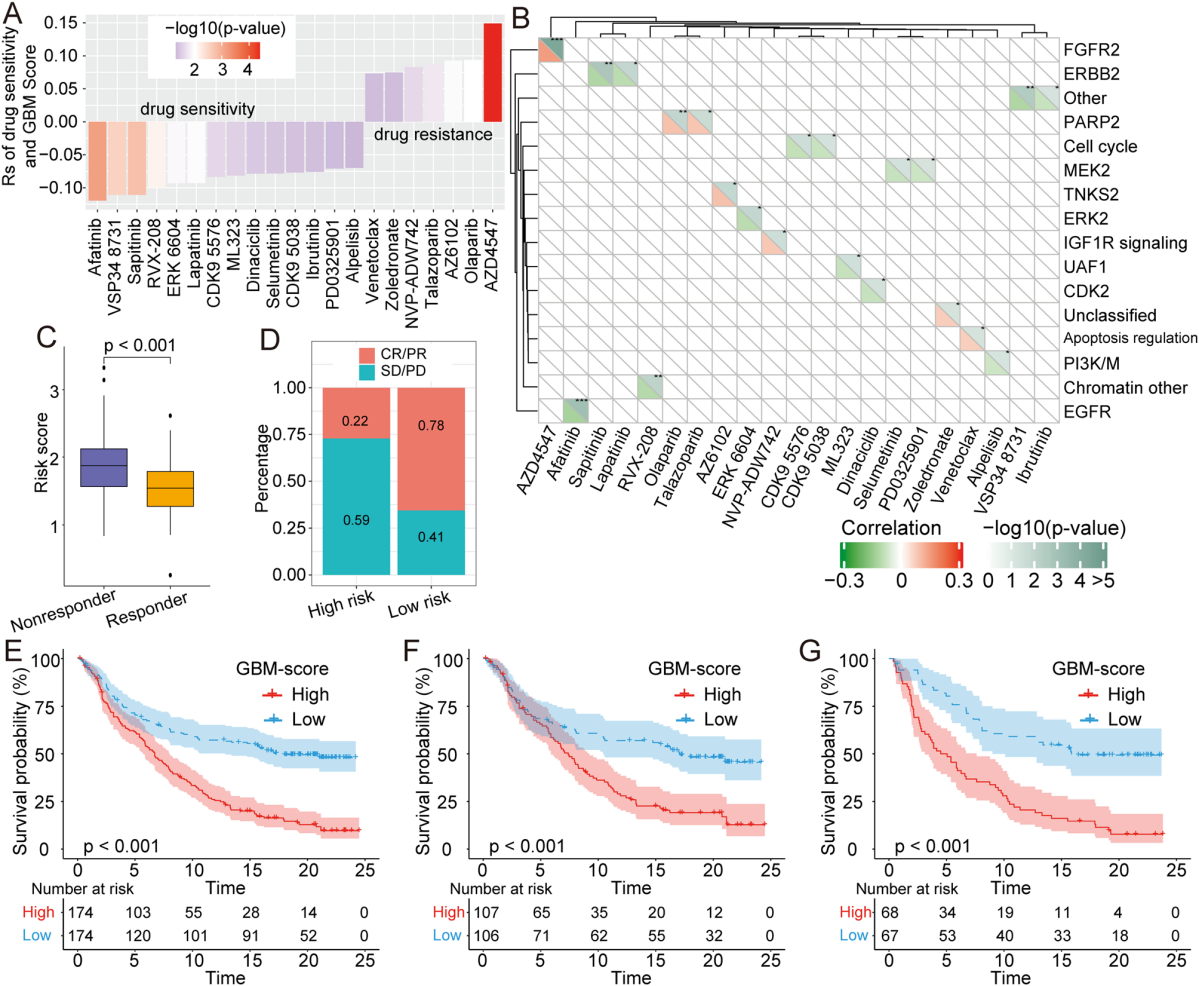
The association between GBM score and drug sensitivity and efficacy of immunotherapy. (**A**) Spearman analysis was used to evaluate the correlation between GBM score and drug resistance. (**B**) The correlation between drugs and signaling pathways targeted by drugs. (**C**) The risk scores between non-responders and responders for anti-PD-L1 treatment in the IMvigor210 cohort. (**D**) Percentage statistics for different degrees of immunotherapy response in low and high GBM score groups. (**E**,**G**) Total patients**,** or Stage I + II samples, or Stage III + IV samples, all showed a significant difference in survival between samples with high and low GBM scores.

Chemotherapy and targeted treatments are therapeutic approaches to GBM^15,16^; however, drug resistance is a major obstacle to the effective treatment of GBM. Therefore, we analyzed the relationship between GBM scores and drug response of tumor cell lines in the Genomics of Drug Sensitivity in Cancer (GDSC) database. We discovered 21 pairs of significant correlations between the GBM score and drug sensitivity (Fig. 6A). There were 14 correlations of drug sensitivity with GBM score and 7 correlations of drug resistance with GBM score. In addition, we determined the signaling pathway genes targeted by these drugs. We found that drugs associated with high GBM scores mainly targeted apoptosis regulation and the FGFR2, PARP2, and IGF1R signaling pathways. In contrast, drugs associated with low GBM scores targeted EGFR, PI3K/M, and cell cycle signaling pathways (Fig. 6B). Thus, GBM scores are not only correlated with drug sensitivity and resistance but may serve as a biomarker that informs clinical decision-making for appropriate treatment strategies.

### Correlation between GBM score and immunotherapy response

Although checkpoint immunotherapy has made a major impact on the treatment of GBM, only a few patients derive a benefit from immunotherapy. Currently, the biomarkers used to predict a response to immunotherapy are the expression of PD-L1 and tumor mutation burden^17–19^; however, the GBM score may predict the response to anti-PD-L1 immunotherapy. Analyzing the IMvigor210 cohort^20,21^, we determined that the degree of response to anti-PD-L1 inhibitors, including complete response (CR), partial response (PR), stable disease (SD), or progressive disease (PD), was linked to GBM scores. The responders had a lower GBM score than non-responders (Fig. 6C). Percentage statistics comparing low and high GBM scores also showed that patients with low scores had significantly better treatment outcomes (Fig. 6D). We compared the proportional distribution of CR/PR/SD and PD in high- and low-risk group patients, and we observed that patients in high-risk group have significantly higher percentage of PD (Fig. S1A), patients with PD have significantly worse overall survival (Fig. S1B). Furthermore, we analyzed the differences in survival statistics for all patients and different stage patients of the IMvigor210 cohort. The results showed that total samples (Fig. 6E), stage I + II of patients with GBM (Fig. 6F), or stage III + IV samples (Fig. 6G) showed a significant difference in survival between samples with high and low GBM scores. In summary, our analysis suggests that RNA modification patterns were closely associated with the response to anti-PD-1/PD-L1 immunotherapy.

## Discussion

In GBM, an invasive human brain tumor with extremely poor survival^22^, RNA modifications play an important role in tumor occurrence and progression^23,24^. Jin et al. reported that the m6A writer WTAP is overexpressed in GBM tissues compared to normal brain tissues. Overexpression of WTAP enhances the proliferation, migration, and invasion of GBM cells, while knockdown WATP mutations have the opposite effect^25^. The m6A writer METTL3 is elevated in glioma stem-like cells (GSCs), where it mediates GSC maintenance and dedifferentiation by regulating SOX2 mRNA stability^26^. Many studies have focused on the role of a single regulator; however, RNA modification is an integrated process involving multiple regulators. In this study, we performed a comprehensive analysis of the role of RNA modification based on the primary regulator writers for two types of RNA modification. Notably, we found that RNA modification patterns correlated with immune cell infiltration and the prognosis for GBM patients.

Tumor heterogeneity contributes to the complex interaction of tumor cells and TMEs, as well as to GBM growth and aggressiveness^27^. Many immune cells, including glioma-associated macrophages (GAMs), CD4 + and CD8 + T lymphocytes, dendritic cells, T regulator cells, and NK cells, have an important role in GBM growth, migration, drug resistance, and response to immunotherapy^27^. GAMs, in particular, correlated strongly with tumor progression and clinical tumor grade^28^. GAMs are involved in remodeling the TME by inducing alterations between the M2 immunosuppressive state and the M1 pro-inflammatory, anti-tumor state. In this study, we found that patients in cluster 1 had worse survival than those in cluster 2. Although the expression plots of M1 and M1 seemed highly heterogeneous in cluster 1 and cluster 2, M2 macrophages tend to higher in cluster 1 than cluster 2, while the M1 macrophages tend to be more enriched in cluster 2. These results suggested that RNA modification patterns are closely correlated with the immune state and prognosis for GBM patients.

High immunosuppression is the primary reason for the failure of immune therapy in GBM patients^29^. While there is no FDA-approved immunotherapy for GBM, ongoing clinical trials, especially for checkpoint inhibitors, show promise for the treatment of GBM. A previous study found that tumor mutational burden and microsatellite instability might predict the therapeutic efficacy of PD-1/PD-L1 inhibitors in GBM patients^29^. Recent studies have revealed that RNA modifications are strongly correlated with the immune microenvironment and the effectiveness of immunotherapy in GBM patients^30,31^. Lin et al. developed a prognostic model based on m6A RNA methylation regulators, which provides a novel biomarker for TME phenotype and GBM prognosis^11^. Xu et al. found that m6A regulators were not only associated with the expression of immune checkpoint genes, such as CTLA-4, B7H3, PD-1, and PD-L1, but also that they predict the efficacy of immunotherapy in GBM^13^. Further, Li et al. established a scoring model based on m6A regulators and TME-related genes that has the potential to predict the efficacy of PD-L1 inhibitors in lung adenocarcinoma^32^. The role of m6A modification in immune regulation and immune response has also been evaluated in colon cancer^12^, gastric cancer^10^, bladder cancer^33^, and head and neck squamous cell carcinoma^34^. Additionally, Chen et al. demonstrated that cross-talk among the main types of RNA modification writers plays a role in TME formation and the efficacy of immunotherapy of colorectal cancer^35^.

Collectively, these results indicate that RNA modification shapes the complexity of TME and may predict the response to immunotherapy. However, the role of many RNA modifications in cancer remains largely unknown. More research in vivo and in vitro is still needed to determine the role of RNA modifications in cancer.

## Conclusions

we performed a comprehensive analysis of the genetic alterations of RNA modification regulators in GMB. The expression of RNA modification regulators was associated with RNA modification patterns and immune cell infiltration in TME. In addition, we develop a scoring model based on the DEGs between clusters showing different RNA modification patterns to predict therapeutic efficacy and prognosis for patients with GBM.

## Methods

### Data collection and processing

Data related to mRNA expression, miRNA expression, gene mutation, copy number variation (CNV), and clinicopathological information of 392 glioblastoma (GBM)) samples were collected from the TCGA database (https://portal.gdc.cancer.gov/). The database platform has obtained ethical approval from all participants. All experiments were performed in accordance with the relevant guidelines and regulations. Data with missing information were removed. 16 of the 167 GBM samples had missing survival follow-up data, so we filtered these samples when we did the prognostic analysis.

After a comprehensive review of the published literature, we identified writer genes for 26 RNA modifications including m6A methylation, APA, A-to-I RNA editing, and m1A methylation. The anti-PD-L1 treatment cohort (Imvigor210) is an immunotherapy cohort for urothelial carcinoma including both clinical and gene expression data^36^, we employed this cohort to assess the relationship between GBM score and immune cell therapy response.

### Unsupervised clustering analysis

We used a consensus clustering algorithm to identify RNA modification patterns based on the expression of the 26 RNA modification regulators as described previously^37^. The optimal number of clusters and their stability were determined by cophenetic, dispersion, and silhouette coefficients using ConsensusClusterPlus^37^. For the principle of the farthest distance between groups, we choose K = 2 as the final number of clusters, according to the smallest clustering within groups.

### Gene set variation analysis (GSVA)

To determine the correlation between biological function and different RNA modification patterns, GSVA enrichment analysis was performed using the “GSVA” R package^38^. The gene sets of “c2.cp.kegg.v7.1” were obtained from the MSigDB database for GSVA analysis. The clusterProfiler R package was used for functional annotation of the 26 RNA modification regulator genes with the cutoff value of false discovery rate < 0.05^39^.

### Identification of cell types by estimating relative subsets of RNA transcripts (CIBERSORT)

To identify immune subsets in the TME, we used CIBERSORT (http://cibersort.stanford.edu/) to determine the abundance of immune cells in GBM. CIBERSORT is a computational method for accurately estimating immune cell composition from tissue gene expression profiles^40^. The final CIBERSORT output estimates were normalized to directly compare cell fractions across different immune cell types. The optimal cut-off values for a fraction for each immune subset were defined as the point with the most significant split and were calculated using the web-based tool “cutoff Finder”^41^.

### Generation of an RNA modification gene model

To quantify the RNA modification patterns for individual patients, we developed a scoring system. Procedures for establishing the gene signature for 26 writers were as follows. DEGs that were associated with different RNA modification patterns were normalized, after which overlapping genes were extracted. An unsupervised clustering method was applied to classify patients into several groups for further analysis. Consensus clustering analysis was used to define the number of clusters of genes and their stability. Then, Cox regression was applied to perform a prognostic analysis for each gene. Significant genes were selected for further analysis, after which principal component analysis (PCA) was used to construct an RNA modification gene signature. We then defined the RNA modification score (GBM score) with a method close to gene expression grade index (GGI):

GBM score = ∑(β_i_ × Exp_i_), here β is the coefficient of the gene in the univariate Cox regression of the corresponding gene and i is the expression of RNA modification phenotype-related genes^20,42^.

### Association between GBM score and transcriptional and post-transcriptional events

We analyzed differences in expression of miRNAs between high and low GBM score groups using the Wilcoxon test. Then, KEGG enrichment analysis was used to identify potential correlations between targeted signaling pathways based on the differential expression patterns of miRNA.

### Statistical analysis

Statistical analysis was carried out using R (version 3.6.1). The Wilcoxon test was used to calculate differences between the two subgroups. Student’s t-tests and one-way ANOVA were used to compare differences among three or more subgroups. Patient prognosis was tested using Kaplan–Meier analysis and the multivariable Cox regression model. Performance of the GBM scores was assessed using a receiver operating characteristic (ROC) curve. A *P*-value < 0.05 was considered statistically significant; * *P* < 0.05, ** *P* < 0.01, *** *P* < 0.001.

### Ethics approval and consent to participate

Data was collected from public databases.

## Supplementary Information

Supplementary Figure 1.

Supplementary Table 1.

Supplementary Legends.

## Data Availability

The data that support the findings of this study are available from the corresponding author upon reasonable request.
