# Supplementary figures and images for: RNA modification patterns based on major RNA modifications define tumor microenvironment characteristics in glioblastoma
Source: Sci Rep. 2022 Jun 18;12:10278. doi: 10.1038/s41598-022-14539-6 (PMC9206649; doi:10.1038/s41598-022-14539-6)

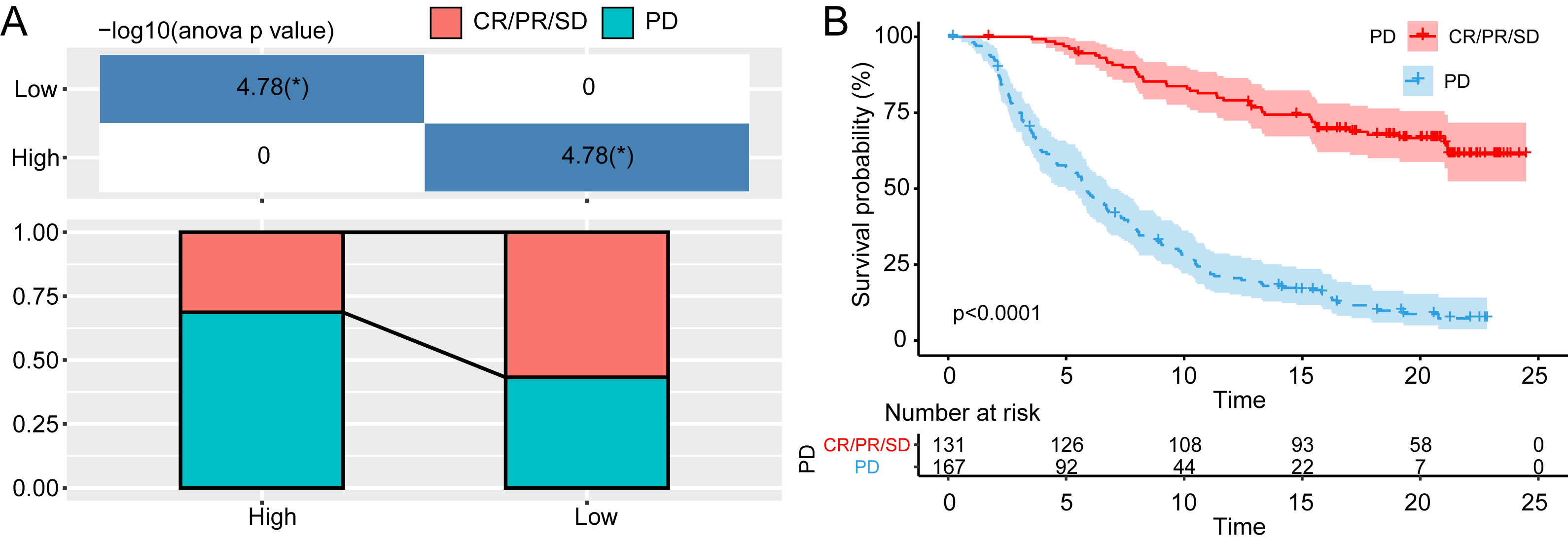

Supplement: Supplementary file 1 — Supplementary Figure 1. [file 41598_2022_14539_MOESM1_ESM.tif]
