# Supplementary material for: RNA modification patterns based on major RNA modifications define tumor microenvironment characteristics in glioblastoma
Source: Sci Rep. 2022 Jun 18;12:10278. doi: 10.1038/s41598-022-14539-6 (PMC9206649; doi:10.1038/s41598-022-14539-6)
Supplement: Supplementary file 2 — Supplementary Table 1. [file 41598_2022_14539_MOESM2_ESM.docx]

**Supplementary Table 1. Summary of RNA modification writers.**

| **RNA modification type** | **Writer** |
| --- | --- |
| m6A | METTL3 |
| m6A | METTL14 |
| m6A | WTAP |
| m6A | RBM15 |
| m6A | RBM15B |
| m6A | ZC3H13 |
| m6A | KIAA1429 |
| m1A | TRMT61A |
| m1A | TRMT10C |
| m1A | TRMT61B |
| m1A | TRMT6 |
| APA | CPSF1 |
| APA | CPSF2 |
| APA | CPSF3 |
| APA | CPSF4 |
| APA | CSTF1 |
| APA | CSTF2 |
| APA | CSTF3 |
| APA | CFI |
| APA | PCF11 |
| APA | CLP1 |
| APA | NUDT21 |
| APA | PABPN1 |
| A-I | ADAR |
| A-I | ADARB1 |
| A-I | ADARB2 |
