# Supplementary material for: RNA modification patterns based on major RNA modifications define tumor microenvironment characteristics in glioblastoma
Source: Sci Rep. 2022 Jun 18;12:10278. doi: 10.1038/s41598-022-14539-6 (PMC9206649; doi:10.1038/s41598-022-14539-6)
Supplement: Supplementary file 3 — Supplementary Legends. [file 41598_2022_14539_MOESM3_ESM.docx]

**Supplementary figure legend**

**Supplementary Fig. 1.** The proportional distribution of different degrees of immunotherapy response CR/PR/SD and PD in high- and low-risk group patients. a. Patients in high-risk group have significantly higher percentage of PD. b. The difference in prognosis between patients with CR/PR/SD and PD.
